# ADAPTING A DEMENTIA CAREGIVER CURRICULUM ACROSS THREE HEALTH PROFESSIONS

**DOI:** 10.1093/geroni/igad104.2420

**Published:** 2023-12-21

**Authors:** Edmund Duthie, Kathryn Denson, Deborah Simpson, Stacy Barnes, Jennifer McAlister, Amanda Szymkowski, Rachel Kavanaugh, Wendy Betley

**Affiliations:** Medical College of Wisconsin, Milwaukee, Wisconsin, United States; Medical College of Wisconsin, Milwaukee, Wisconsin, United States; Advocate Aurora Health, Milwaukee, Wisconsin, United States; Marquette University, Milwaukee, Wisconsin, United States; Alzheimer’s Association, Milwaukee, Wisconsin, United States; Medical College of Wisconsin, Milwaukee, Wisconsin, United States; Medical College of Wisconsin, Milwaukee, Wisconsin, United States; Alzheimer’s Association,, Milwaukee, Wisconsin, United States

## Abstract

Unpaid caregivers provide >80% of the care for the 6.2 million Americans living with dementia, yet healthcare professionals rarely refer them to community services. The Alzheimer’s Association’s “Direct Connect” (DC) referral program is provider-initiated connecting patients/caregivers with resources to improve care and quality of life. Our interprofessional team created a 15-minute curriculum to educate students about DC and how to advocate for DC use at clinical sites. Champion faculty, from each profession, utilized a common presentation, with minor modifications for professional relevance. Delivery was both synchronous/asynchronous to learners from 2 different universities with knowledgeable clinician(s) available to answer questions. A Qualtrics survey collected feedback (N=218; 105 medicine, 57 nursing, 56 pharmacy). Relevance of the curriculum to each of the 3 professions was high (1-5 scale; mean range 4.46 – 5.00), with nursing rating it as most relevant. Feedback was also quite positive across the professions (78%-92% rated the curriculum quality as excellent/very good overall), with nursing giving the highest ratings. Most learners indicated a strong likelihood of using DC in clinical settings; 64-100% are very likely or somewhat likely to do so, with nursing being most likely to make referrals. In summary, a common dementia DC curriculum was successfully implemented for learners in 3 health professions; it was highly relevant to each profession, well accepted, and perceived to be clinically actionable. Nurses tended to be the most positive on these measures. While these results are promising, DC remains underutilized and initiatives to further increase referrals from providers must be expanded.

